# Imposter Syndrome: A Reflective Discourse into the Experiences of Canadian Black Nurses through Art

**DOI:** 10.1177/08445621241289727

**Published:** 2024-10-17

**Authors:** Nadia Prendergast, Ola Abanta Thomas Obewu

**Affiliations:** 1 Daphne Cockwell School of Nursing (DCSN), 7984 Toronto Metropolitan University, Toronto, ON, Canada

**Keywords:** Black nurses, imposter syndrome, anti-Black racism, reflective discourse, afrocentric*, art-informed

## Abstract

Impostor syndrome is a common phenomenon experienced by individuals when entering new ranks in the workplace. Although women experience greater feelings of imposter syndrome than men, Black individuals report a prolonged experience of imposter syndrome when compared to their white counterparts, which negatively impacts their everyday experiences, health, and overall well-being. With growing studies showing the pervasive nature of anti-Black racism on the health of Black people, there remains a paucity of studies showing the connection between anti-Black racism and imposter syndrome. Within nursing, anti-Black racism can be seen to foster imposter syndrome through discriminatory practices that affect the career development, recruitment, and retention of Black nurses. Anti-Black racism is prevalent, and, in this paper, two Black nurses share insight through their own encounters with imposter syndrome and its relationship with anti-Black racism. Guided by Black feminist thought, they use art to navigate their reflective discourse as a means of reclaiming their identity and positionality as leaders in their rights. Reflective discourse is ideal for transformative learning to occur through dialogue. In addition, it promotes the use of art for deeper discussions when understanding the Black experience. Together, these nurses explicate how adopting Afrocentric knowledge and practices through their reflective discourse can affirm their identity, promote a sense of belonging, and assist in dismantling the effects of anti-Black racism and imposter syndrome within nursing.

Imposter syndrome (IS) is a prevalent phenomenon experienced by most individuals entering new positions or ranks in the workplace across diverse professions. IS is defined as a condition in which an individual fails to internalize their accomplishments and successes due to a constant feeling of self-doubt, deficiency, and intellectual fraud (Bravata et al., 2020; Clance & Imes, 1978; Clark et al., 2022). Individuals who experience IS struggle to accredit their achievements and performance to their intellectual capabilities, and fear being exposed as fraud. Earlier conversations on IS centred on binary gendered differences where women were significantly impacted by IS than men when occupying similar work positions (Clance & Imes, 1978; Cokley et al., 2015; Cusack et al., 2013). Gradually, these conversations progressed toward race and its outcome on Black individuals in their careers, academic journeys, and health (Bernard et al., 2017; McClain et al., 2016; Meadhbh Murray et al., 2022). Although IS is common, it manifests differently within the Black experience (Bernard et al., 2018; McClain et al., 2016). Black individuals report a prolonged experience of IS when compared to their white counterparts, which extensively harms their personal lives, work experience, and overall health (McClain et al., 2016; Meadhbh Murray et al., 2022; Stone et al., 2018). There continues to be a scarcity of data on how IS operates when gender and race intersect (Bernard et al., 2017; Crenshaw, 1989; Hall et al., 2012). One area is the gendered-dominated profession of Canadian nursing; In 2021, approximately 91% of Canadian registered nurses were female (Canadian Nurses Association [CNA], n.d.). However, within the scholarship of nursing, there remains a lack of race-related data (Registered Nurses’ Association of Ontario [RNAO], 2022). Ongoing studies have demonstrated the perpetual relegation of Black and racialized nurses to bedside positions while possessing comparable and competitive qualifications as their white counterparts (Cooper Brathwaite et al., 2022b; Iheduru-Anderson, 2020; Premji & Etowa, 2014). Such normalized practices have been aligned with racism and identified as the Cappuccino principle, where white nurses occupy leadership positions at the top and Black and racialized nurses occupy bedside positions at the bottom (De Sousa et al., 2023; Jacobs, 2007).

Anti-Black racism functions invisibly to foster elevated feelings of IS in Black nurses, while gatekeeping Eurocentrism in nursing. This shared experience of IS, due to discrimination and stereotypes, amongst Black nurses leads to poor health outcomes of chronic health conditions brought about by stress, feelings of alienation, and not feeling a sense of belonging (Clarke, 2022; Ford et al., 2021; Robinson, 2014). Resulting in the loss of identity, burnout, and a decline in overall health and well-being, IS may have a significant impact on the recruitment and retention of Black nurses considering international and national recruitment strategies (Health Canada, 2023; International Council of Nurses, 2021; Silas, 2012; World Health Organization, 2020). The need for research and data is critical, but equally important are the voices of Black nurses to be heard if we are to gain an understanding of the operational nature of IS within their experiences.

In this paper, two Black nurses, one a novice nurse and the other a Black assistant professor, share insights into the continuum nature of IS within the Black experience. A recent study by Junious (2024) demonstrates an ongoing relationship between IS and the historical, systemic barriers in the experiences of African American faculty. As a Black novice nurse and a Black faculty working within Canada, we suggest that a reflective discourse can explore how IS functions within a Canadian context. Reflective learning is central to nursing education (College of Nurses of Ontario, n.d.), therefore, beginning our dialogue within the reflective discourse allows us to heal and flourish within the nursing profession. Implementing Black feminist thought (BFT) to navigate our reflections, we interrogate and reclaim our identity and positionality as leaders using an art illustration (see Figure 1). Furthermore, we explicate how resistance to anti-Black racism is made possible by adopting Afrocentric knowledge and practices to affirm our identity, promote a sense of belonging, and work towards restoring our health and well-being.

**Figure 1.**
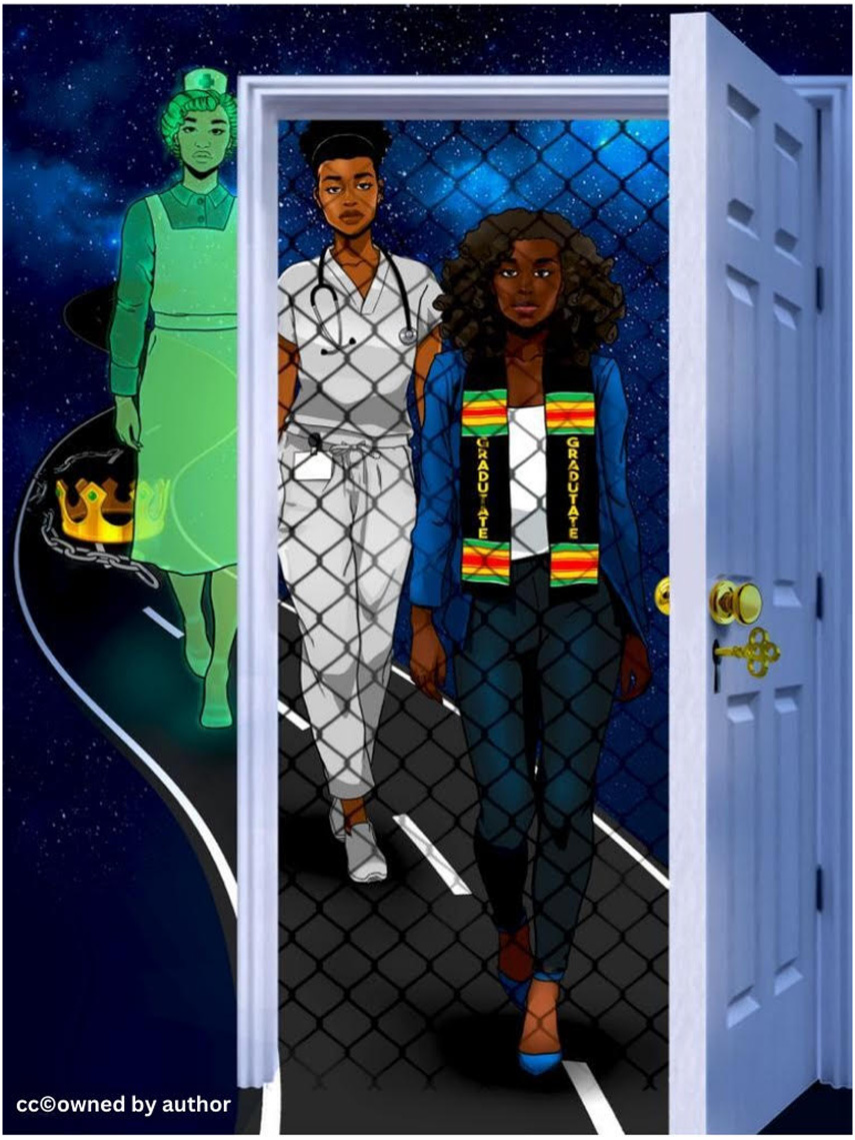
Imposter Syndrome.

## Anti-Black racism within Canadian nursing

The existence of anti-Black racism and discrimination in Canada has often gone unchecked until 2020, when the COVID-19 pandemic exposed the social and health equities embedded in our society with several racially induced killings, including the death of George Floyd, which raised a global uproar. CNA (2020) released a statement labelling anti-Black racism as a public health emergency in Canada. This was supported by a report by the Public Health Agency of Canada (2020) declaring anti-Black racism as a health crisis and a key driver within the Social Determinants of Health. Black advocate groups and nursing organizations have pointed out the lack of Black-related data in nursing (Jefferies et al., 2022b; Onagbeboma, 2020; RNAO, 2022). Although healthcare organizations continue to denounce anti-Black racism and publicize measures to be taken to dismantle it, active and practical steps within Canadian nursing seem to be lagging. This delayed response continues to have a toll on the mental well-being of Black nurses who constantly experience microaggression and multiple forms of racism (Byers et al., 2021; Iheduru et al., 2021).

Nurse education remains silent on the historical events that excluded Black nurses from entering nursing in Canada. Like the school segregation system of the United States, Canada has also been complicit in such practices, where Black nurses were segregated through racist policies and prohibited from enrolling in nursing schools until 1948 (Flynn, 2009; Jefferies et al., 2022a). Today, such beliefs and practices remain embedded within nursing, where the white middle-class nurse is considered as the ideal type (De Sousa et al., 2023; Jefferies et al., 2022a; Prendergast, 2014). It is within this constructed environment that IS exists, providing a unique yet disturbing experience for two Canadian Black nurses, a novice nurse, and an assistant professor. In this paper they share their journey of IS through their reflective discourse and the use of art where they provide deeper insight into how they arrive at their healing and restoration.

## The reflections of a black novice, bedside nurse

I always pride myself as an African woman, Nigerian to be precise. Growing up in Nigeria signified identity awareness and an in-depth recognition of community and guidance. The cliché of “I didn’t know I was Black till I migrated to Canada” remains true and resonates with me. My journey in nursing school started during the period when I was completing the requirements needed for my BScN program. I vividly recall an encounter which the school counsellor tried to dissuade me from enrolling in the BScN program, even with the explicit declaration of my desire to attend medical school. To put this into context, a degree certification is a primary requirement for medical schools. Yet, the school counsellor deemed it more appropriate to direct me towards a nursing diploma, claiming it would be “easier and faster” for me. Unfortunately, academic streamlining is not uncommon for Canadian Black students, even when they have shown exceptional intellectual capabilities (James, 2019; 2020).

Academic streamlining perpetuates anti-Black racism within the experiences of career development among Black people, leading to a continual absence of Black people in leadership and policy-making positions, and reinforcing exclusionary practices (James & Turner 2017; Lopez & Jean-Marie, 2021). Unless Black nurses have support to move beyond the barriers brought about by academic streamlining, the underrepresentation of Black nurses will remain. Fortunately, I was encouraged by my Nigerian community to reject the narrative and continue with my academic upgrade for a BScN certification. Afrocentrism is founded on community collectiveness (Bay, 2000; Lateef & Balakrishnan, 2023), and it was a support network that became central to my navigating and advocating for my rights. However, I have always pondered on how other Black people have been streamlined into a diploma program when they could achieve beyond.

My first two years in nursing school were not as psychologically seamless as they appeared to be. I felt like an outsider and was convinced that the nursing profession was not for me, mainly due to the lack of representation in leadership positions. A systematic review by Osakwe and colleagues (2022) highlights that when nursing students have mentors or see individuals with similar identities to theirs in leadership roles, it has a positive effect on their confidence and aspirations. Therefore, the lack of representation would limit my opportunities for a mentor who could support my career and academic development (Luhanga et al., 2023). Despite achieving good grades and the occasional encounters with a Black clinical instructor, I always felt I was never good enough, a compounded feeling of being an imposter.

IS is a debilitating experience for Black novice nurses. The drive to be the “ideal’ is often tainted with noticeable differences such as my hair, skin, and accent, which resulted in me internalizing the microaggression from colleagues and patients that I was a fraud, and did not belong in this ideal, white space (Bell, 2021; Cox et al., 2021). IS has been associated with anxiety and self-blame, and each of these played out in my experience within the classroom, and later in the workplace. I found myself constantly working on proving myself while having to remain invisible and silent within a perceived caring profession (Byers et al., 2021; Luhanga et al., 2023). I was incapable of processing the multiple forms of racism that played out in horizontal bullying and power dynamics within nursing leadership. Relating to my experience as a Black novice nurse, Mitchell (2020) proposes that Black novice nurses are more susceptible to leaving the profession due to unsupportive work environments and the toll of anti-Black racism on their mental health. In addition, a study by Prendergast and friends (2023) revealed that Black nurses were more likely to be harshly disciplined on small issues when compared to their white counterparts who performed serious offenses. As such, being constantly aware and afraid of these events has perpetuated IS within my experience as a Black novice nurse and the toll it has not only on my health but also on my identity.

My experience of Black mentorship and representation became my source of sustenance in my last years as a student and now, as a Black novice nurse. In 2020, the death of George Floyd and the axiom, “I can’t breathe” rang true to the experiences of a plethora of Black nurses with whom I could identify. Through RNAO's Black Nurses Task Force (BNTF), where I sat as a student panel member among other phenomenal Black nurses, I was able to breathe and be empowered to speak where others could not. The tragedy of the COVID-19 pandemic, the death of George Floyd, and protests of the Black Lives Matter Movement became a catalyst by which I could redefine my identity, challenge IS, and engage with other Black nurses who were sharing the same experiences of IS as myself. I have, since then, realized I was not alone. I share a common space and experience with other Black nurses who provide validation, hope, affirmation, and belonging.

## The reflections of a black nursing professor assistant

O Canada! We stand on guard for thee! The words of the Canadian anthem had sentimental meaning for me as a Black nurse, born in England to Caribbean parents who struggled and were never recognized for their contributions to the success of first-world countries such as England (Government of United Kingdom, 2017). Coming to Canada was the ideal image of a multicultural society that supported the flourishing of all people (Prendergast, 2014). But to my surprise, I encountered a new form of racism that was obscure, invisible, yet pervasive and this played out in sustaining the white settler space of leadership and the relegating and re-naming all other non-whites as the workers and reserved army (Bannerji, 1997). As a Black nurse educated in the United Kingdom, navigating my way into foreign spaces began a series of experiences of feeling that I was an Imposter. To survive the various spaces and their toll on the Black body, with studies showing that Black faculty are underrepresented and often leave after a short while, I have had to understand the barriers in place that result by identifying three processes within my experience which are: the mentoring process, assimilating to the status quo, and the taxation on the Black body (Cooper Brathwaite et al., 2022b; Iheduru-Anderson et al., 2022b).

The mentoring process for faculty is ongoing where we work in partnership with senior faculty in carving out our program of research, but the lack of Black representation and the sparse interest within nursing in social justice issues is strongly reflected within nursing education (Iheduru-Anderson et al., 2022b; Mokel et al., 2022). This required me to seek out the few senior faculty members who had an interest in addressing racism, specifically anti-Black racism within nursing. The mentorship experience became twofold, where I, as a Black professor, was mentoring students and, at the same time, relying on the few faculty members who were willing to mentor me. White critical allies play an important role in the mentoring of Black faculties, and their work often goes unnoticed by other senior faculty members who fail to support them. Therefore, the mentorship role for Black professors becomes equally heavy and lonely, as depicted in Whitfield-Harris et al. (2017), where the pressure to assimilate to the status quo is considered easier and less provocative (Beagan et al., 2023; Iheduru-Anderson et al., 2022a).

Equity, Diversity, and Inclusion (EDI) has been criticized as being a form of assimilation, leading to the invisibility and misrepresentation of anti-Black racism practices (Iheduru-Anderson et al., 2022b). Black nurses are frequently asked to sit on such committees hoping to navigate change and be a voice for Black people. Still, while there, we find ourselves compelled to assimilate into a space that favours other oppressive issues and refrains from challenging the urgency of anti-Black racism (Prendergast et al., 2023).

The taxation on the Black body has been well documented, showing how Black faculty are burnt out, overwhelmed, and again, face barriers as their area of research on race is often met with unjustified criticisms by their peers (Beard & Julion, 2016). Such terms as “your language is too harsh” or maybe watering down what you are saying is a result of white fragility at play (DiAngelo, 2018). Many Black professors struggle in publishing within nursing journals, and this has a direct impact on students being aware of Black scholars (Iheduru-Anderson et al., 2022a; Mkandawire-Valhmu et al., 2010; Social Sciences and Humanities Research Council of Canada, 2021). As a Black professor, I am faced with the undermining of my abilities, questioning of my credentials, and the continual fear of being reported by students who fail to see me as equal to my white colleagues. Studies by Beard and Julion (2016) and Whitfield-Harris et al. (2017) illustrate that Black faculty were more likely to be criticized by white students, consequently impacting their self-esteem. The lack of support by colleagues in refraining to call out the wrong or remaining silent taxes the body of Black professors, resulting in weathering and allostatic load (Iheduru-Anderson et al., 2022a; Guidi et al., 2021).

Following the COVID-19 pandemic, I have had to rethink and revisit the world as I know it. Students who are not exposed to anti-Black racism are asking questions and from a generation who speak through cancel culture and calling out, I realize that IS can be challenged within the context of nursing education. In my class, I not only challenge anti-Black racism but also find healing through art. Supported by Black feminist thought and art, this allows me to create a classroom space that is culturally responsive to the diversity and experiences of my students (Du Bois, 1926; Lapum & Hume, 2015). One such way is the use of digital illustration (See Figure 1).

## Harmonizing our reflections

The interruption of slavery within the Afrocentric culture relies on the connection between BFT and Afrocentricity to explain the Black experience. BFT examines the intersection of race, class, and gender within the marginalization of Black women in society (Collins, 1989). Meanwhile, Afrocentricity, according to Asante (1980), is defined as a theory that centres the experiences of people of African descent within the context of their historical, cultural, and social locations. Therefore, as a Black novice nurse and a Black faculty member, these theories centre our experiences and provide a meaningful understanding of the nuances of Blackness that are “invisible to the wider white society” (Government of Ontario, 2024). We use BFT and Afrocentricity throughout our reflective discourse to expose the continuum nature of IS.

Mezirow (1990) refers to reflective discourse as a dialogue that leads to experiential learning. Using reflective discourse as our basis for understanding IS, we encountered diverse emotions brought about by historical, social, and political circumstances. However, it was through our insight of BFT and Afrocentricity that we identified our shared adversities and harmonized our experiences which led to an art illustration (see Fig 1). As a result, the art illustration is the outcome of our reflective discourses and journey into understanding IS.

## Art - a path to healing and resistance

Afrocentric knowledge and values are formidable tools in reconciling Black individuals to their roots and identity (Sefa Dei, 1996; Wane & Todd, 2018). By embracing Afrocentricity, a unique elder-student relationship is formed between the professor and novice nurse through their reflective discourse and the use of art (Sefa Dei, 2012; Khalifa, 2018; Mezirow, 1990). The art of nursing is commonly associated with care and compassion but fails to represent racism and oppressive issues that lurk within its practice (Cooper Brathwaite et al., 2022a; Prendergast et al., 2023). By incorporating the works of Hooks (1996) and Du Bois (1926), we redefine art as a space of resistance that promotes our healing, care, and compassion. We use art as a critical language to redefine our journey as Black nurses. According to Du Bois (1926), the use of art is a space where one's thoughts can be expressed without being judged. We find commonalities in our voices as a means of unveiling our lived realities and countering the inaccurate, dominant narratives of what it means to be Black nurses and women (De Sousa et al., 2023).

Art provides a space by which a convergence of solidarity occurs between the professor and novice nurse. BFT depicts the works of Black feminists such as Collins and Hooks who connect art with the Black identity and the preservation of Black legacy (Collins, 1989; Hooks, 1996). Here, we implement counter-storytelling and center our voices to be heard (Pender et al., 2023). The art depicts our journey of the past, present, and dreams of the future. From the illustration (Fig 1), we are conscious that our history begins with the crown, signifying our civility and royalty, and debunking the false narrative that our existence began with the Trans-Atlantic slave trade. Together, we journey through the invisibility of a green translucent nurse, demonstrating how Black nurses were barred from nursing in Canada as late as 1948. Embracing the words of Mya Angelou, we reverberate through our illustration, “Yet, still [we] rise” (Angelou, 1978; Collins, 1989). Our resiliency of rising above the socio-political barriers is depicted in the constant march for success, despite the invisibilities we face. As policies and statements are instrumental in promoting equity and opportunity we visualize them as an illusive open door in our illustration, where the wider white society fails to see the netted fence that impedes our advancement through the notion of IS. Our reflective discourses create critical dialogues where we ask questions, share our wisdom, find healing, and experience restoration through art.

## Implications and conclusion

Our reflections on IS within Canadian nursing unmask IS to be a vehicle for which anti-Black racism can persist and lead to adverse impacts on our health and identity as Black nurses. While we have converged and forged a space of resistance and healing through reflection and art, anti-Black racism continues to operate densely and imperceptibly through IS to sustain the invisibility of Black Canadian nurses. Hence, we add our voices with Black and allied advocates that call for actionable measures to dismantle anti-Black racism within Canadian nursing, while fostering a space of belonging for Black nurses to advance professionally. We recommend further studies that focus on the experiences of Black nurses and the impact of IS on their health and career development. To appreciate the experiences that Canadian Black nurses face, race-based data is required to inform EDI policies that challenge anti-Black racism and improve the retention of Black nurses. During our reflection, we fostered a unique elder-student relationship rooted in mentorship which enhanced our sense of belonging and identity. For this reason, we recommend mentorship programs that incorporate Afrocentric values and are tailored for Black nurses. We conclude that through reflective discourse, the use of art, and the application of Afrocentric values, we can interrogate IS and create a path of resilience, determination, and community responsibility that can restore our health and well-being.
